# Autocrine Sonic Hedgehog Attenuates Inflammation in Cerulein-Induced Acute Pancreatitis in Mice via Upregulation of IL-10

**DOI:** 10.1371/journal.pone.0044121

**Published:** 2012-08-30

**Authors:** Xiangyu Zhou, Zhiqiang Liu, Feng Jang, Chuannan Xiang, Yuan Li, Yanzheng He

**Affiliations:** 1 Department of Vascular Surgery, Affiliated Hospital of Luzhou Medical College Luzhou, Sichuan, China; 2 Department of Lymphoma and Myeloma, Division of Cancer Medicine, Center for Cancer Immunology Research, The University of Texas MD Anderson Cancer Center, Houston, Texas, United States of America; 3 Department of Cardiology, Affiliated Hospital of Luzhou Medical College, Luzhou Sichuan, China; 4 Department of General Surgery, Luzhou People's Hospital, Luzhou, Sichuan China; 5 Institute of Digestive Surgery, State Key Laboratory of Biotherapy, West China Hospital, Sichuan University, China; University of Navarra, Spain

## Abstract

Hedgehog signaling plays critical roles in pancreatic oncogenesis and chronic pancreatitis, but its roles in acute pancreatitis (AP) are largely ambiguous. In this study, we provide evidence that Sonic hedgehog (Shh), but neither Desert hedgehog (Dhh) nor Indian hedgehog (Ihh), is the main protein whose expression is activated during the development of cerulein-induced acute pancreatitis in mice, and the Shh serves as an anti-inflammation factor in an autocrine manner. Blocking autocrine Shh signaling with anti-Shh neutralizing antibody aggravates the progression of acute pancreatitis. Mechanistic insight into Shh signaling activation in acute pancreatitis indicates that inflammatory stimulation activates Shh expression and secretion, and subsequently upregulates the expression and secretion of interleukin-10 (IL-10). Moreover, inhibition of Shh signaling with neutralizing antibody abolishes IL-10 production in vivo and in vitro. Molecular biological studies show that autocrine Shh signaling activates the key transcriptional factor Gli1 so that the target gene IL-10 is upregulated, leading to the protective and anti-inflammatory functions in the mouse model of acute pancreatitis. Thus, this study suggests autocrine Shh signaling functions as a protective signaling in the progression of acute pancreatitis.

## Introduction

Acute pancreatitis (AP) is one of the most common inflammatory diseases with a wide spectrum of severe complications and significant mortality, which affects millions of patients each year in the world [1]. Acute pancreatitis results from acute inflammatory injury of the pancreas, and once the process is initiated, the severity is largely determined by the activated inflammatory response, and it is this systemic response that is believed to be ultimately responsible for most mortality [2]. Despite the increasing research in the latest decades, the pathophysiological mechanisms responsible for the pancreatic inflammatory are not thoroughly elucidated, let alone any effective treatments. A better understanding of the molecular and signaling pathway involved in the series of events leading to pancreatitis could eventually lead to novel preventive and therapeutic strategies.

The Hedgehog (Hh) protein family is a group of secreted intercellular signaling molecules that are essential for cell fate and patterning during the development of liver and pancreas [3]. Loss- and gain-of-function studies in mice have revealed that deregulation of Hh activity affects pancreas morphogenesis and function [4], [5], [6]. In mammals, three proteins constitute the Hh family: Sonic hedgehog (Shh), Indian hedgehog (Ihh), and Desert Hedgehog (Dhh) [7]. Shh is the best studied of mammalian Hhs with the broadest expression pattern, including in the developing nervous system, limb buds, skin and gut. Ihh expression is restricted to the developing bone and cartilage, gut and pancreas, whereas Dhh expression is found primarily in the gonads and testes, with some expression also in peripheral nerves and pancreas [8], [9], [10]. All Hh proteins share a common signaling pathway, where Patched (Ptch) and Smoothened (Smo) are the membrane receptors. In the absence of ligand, Ptch exerts an inhibitory effect on Smo activity that is abrogated after Hh binding. In the absence of Ptch, Smo is constitutively active [11]. At the end of the Hh signaling pathway are the members of the Gli family of transcription factors, Gli1, Gli2, and Gli3. In them, Gli1 and Gli2 are considered as positive regulators, whereas Gli3 is mainly function as a negative regulator of transcription, respectively [12].

Recently emerging studies revealed that perturbation in Hh proteins and signaling pathway play an important role in congenital disorders and adult pancreatic diseases, such as pancreatic cancer, chronic pancreatitis, and pancreas regeneration, because they are involved in the early pancreatic development, determination the fate of the mesoderm of the gut tube, islet cell function, pathogenesis and progression of pancreatic cancer and of chronic pancreatitis [13], [14]. Furthermore, latest experiments showed that hedgehog signaling is also important in acute injury and inflammation [15], [16]. However, whether Hedgehog signaling plays any role in acute pancreatitis and the detailed function as well as the mechanisms are poorly studied, regardless a newly published evidence discovered that Sonic hedgehog expression was activated in the cerulein-induced pancreatitis [17].

Based on the above considerations, we undertook the current study to investigate the functions of Hh signal on the acute pancreatic inflammation in vivo and in vitro, and to identify the possible role of Hh signal in the pathogenesis of the disease. Our data showed the expression of Shh, but neither Dhh nor Ihh was dominantly increased in the cerulein-induced acute pancreatitis in mice, and the Shh served as an anti-inflammation factor in an autocrine manner. Blocking autocrine Shh signaling with anti-Shh neutralizing antibody aggravated the progression of acute pancreatitis. Mechanistic insight into Shh pathway activation in acute pancreatitis indicated that inflammatory stimulation activated Shh expression and subsequently upregulated the expression and secretion of IL-10, whereas inhibition of Shh with neutralizing antibody abolishes IL-10 production in the cerulein-induced acute pancreatitis in mice and in the pancreatic acinar cells. Molecular biological studies showed that autocrine Shh signaling activated the key transcriptional factor Gli1 so that the target gene IL-10 was upregulated, leading to the protective and anti-inflammatory functions in the mouse model of acute pancreatitis. Our study suggests that autocrine Shh signaling functions as a protective signaling in the progression of acute pancreatitis.

## Materials and Methods

### Animals Grouping and Acute Pancreatitis Induction

Male C57BL/10SnJ mice of 6–8 weeks old and 22–24 grams in weight used in this study were supplied by the Experimental Animal Center of Luzhou Medical College (Luzhou, China). Mice were housed in a specific pathogen-free environment and maintained at 23°C on a 12-hour light/dark cycle and starved for 12 hours before experimentation except free access to water. All the animal experiments were approved by The Animal Care and Welfare Committee of Luzhou Medical College, and conducted according to the guidelines of the Local Animal Use and Care Committees of Luzhou as well as the National Animal Welfare Law of China.

The acute pancreatitis mouse model was induced as previously described [18]. In brief, the acute pancreatitis was induced by an intra-peritoneal injection of cerulein (Sigma, MO) at a dose of 50 µg/kg body weight at hourly intervals for 7 times; timing was made after the last injection, and sampling was made at the 0, 8th, 16th, and 24th hour. The control mice were treated with the same volume of PBS. Peripheral blood was collected via mice tail vein and the serum was harvested after high speed centrifuge. The control IgG or mouse neutralizing antibody were administrated together with cerulein via intravenous injection.

### Cells, Transfection, and Reagents

The AR42J cells were purchased from ATCC (Rockville, MD), and cells were cultured in RPMI-1640 supplemented with 10% fetal bovine serum plus penicillin (100 U/ml) and streptomycin (100 µg/ml) at 37°C in a humidified 5% CO_2_ atmosphere. For transient transfections of AR42J cells, Lipofectamine 2000 (Invitrogen,CA) was used according to the manufacturers’ protocols.

The anti-mouse Shh neutralizing antibody was purchased from Sigma Aldrich Biotechnology (Sigma Aldrich, MO), the Shh antibody for western blot, sc-365112 Shh (E-1), was purchased from Santa Cruz (Santa Cruz Biotechnology, CA), and the Shh antibody for Immunohistochemistry was from Millipore (Millipore, MA). The recombinant Sonic Hedgehog/Shh, N-Terminus (461-SH-025) was purchased from R&D systems (R&D Systems, MN). The IL-10 (2G101H7) Antibody for western blot, sc-57245, was purchased from Santa Cruz (Santa Cruz Biotechnology, CA). The MISSION® siRNA Gli1plasmid was from Sigma Aldrich, and the Shh shRNA Plasmid (sc-77337-SH) was purchased from Santa Cruz Biotechnology. The pJT4-Shh and the pcDNA-Gli1expression plasmids were from Dr. Micheal Naski, Department of Pathology in University of Texas Health Science Center at San Antonio.

### Isolation of Dispersed Primary Pancreatic Acinar Cells

Dispersed mouse pancreatic acinar cells were isolated by a modified collagenase digestion procedure according to previously reported reference [19]. Briefly, pancreas was dissected and gently grinded in a 70 µM Nylon cell strainer (BD, NJ) with 4 ml of digestion solution (0.5 mg/ml type IV collagenase in RPMI 1640 medium), and transferred to a conical tube. The suspension was shaken by hand in a 37°C water bath for 5−10 min, after washed twice with RPMI 1640 the acinar cells were cultured in RPMI 1640 supplemented with 10% fetal bovine serum plus penicillin (100 U/ml) and streptomycin (100 µg/ml) at 37°C in a humidified 5% CO2 atmosphere. The whole procedure was performed at room temperature and took about 30 min.

### Real-time Polymerase Chain Reaction (PCR)

Total RNA was isolated from fresh tissue or cells using Trizol reagent (Invitrogen, CA), and an aliquot of 1 µg of total RNA was subjected to reverse transcription using a SuperScript II (Invitrogen, CA) reverse transcriptase PCR kit; 1µl of the final cDNA was applied to real-time PCR amplification with SYBR Green using a StepOne Plus real-time PCR system (Applied Biosystems, NY); and the cDNAs were amplified using the primers listed in Table 1.

**Table 1:** Real-time PCR primers used in this study.

Genes	Direction	Sequence	Length
*Shh(m)*	Forward	TTCTGTGAAAGCAGAGAACTCC	112 bp
	Reverse	GGGACGTAAGTCCTTCACCA	
*Shh(r)*	Forward	AGTGGACATCACCACGTCTG	139 bp
	Reverse	CACCGAGTTCTCTGCTTTCA	
*Dhh(m)*	Forward	GGGACCTCGTACCCAACTAC	90 bp
	Reverse	CTTTGCAACGCTCTGTCATC	
*Dhh(r)*	Forward	TTCAAGGATGAGGAGAACAGC	58 bp
	Reverse	GCTCTTTGCAACGCTCTGT	
*Ihh(m)*	Forward	TTGCCTACAAGCAGTTCAGC	142 bp
	Reverse	CGTCCTTGAAGATGATGTCG	
*Ihh(r)*	Forward	TCACTGGCCATCTCTGTCAT	111 bp
	Reverse	GCGGCCCTCATAGTGTAAAG	
*Ptch1(m)*	Forward	GGTGGTTCATCAAAGTGTCG	145 bp
	Reverse	GGCATAGGCAAGCATCAGTA	
*Ptch2 (m)*	Forward	TTGCACCTTTACTGCTCCAG	97 bp
	Reverse	TGTACCAAGGTGGCTCCATA	
*Smo (m)*	Forward	GCAACAAGTATGGGTTGCTG	87 bp
	Reverse	CGCCAGTGAGTTATCAGCTT	
*Gli1 (r)*	Forward	GGGACTATGTGCTATGCCAA	131 bp
	Reverse	AGTGCCAAACATGGCAAATA	
*Il-10 (r)*	Forward	CCCAGAAATCAAGGAGCATT	129 bp
	Reverse	TCATTCTTCACCTGCTCCAC	
*Gapdh(rodent)*	Forward	TCGGACCACCATTGTGATAG	164 bp
	Reverse	CATTTCCTGCTGTCTGCATT	

Primers used in the current study were designed with the online oligo/primer tools from www.genescript.com, and all the primers are across the exon and intron junctions without cross-reaction with other species except genes only having one exon.

### Western Blot

Western blot was carried out as previously described [20]. Briefly, fresh tissue or cells were lysed using 1×lysis buffer (Cell Signaling Technology, CA), and 50 µg of total protein was separated via electrophoresis on a 4–12% gel (Invitrogen, CA). The gel was then transferred onto a nitrocellulose membrane, immunohybridized with primary antibodies (1∶200) at 4°C overnight, and incubated with 2^nd^ antibodies (1∶1000) at room temperature for 1 hour. After washed, the immunoblot was developed using a chemiluminescence substrate (Thermo Scientific, MA).

### Immunohistochemistry

Paraffin-embedded sections were deparaffinized and blocked, incubated with 1∶200 anti-Shh antibody at 4°C overnight, and then incubated with horseradish peroxidase-conjugated secondary antibody at 1∶500 for 1 hour at room temperature. The sections were developed using a 3,3′-diaminobenzidine tetrahydrochloride (DAB) substrate kit (Thermo Scientific, MA) at room temperature for 1–5 minutes and then counterstained with hematoxylin.

### Enzyme-linked Immunosorbent Assay (ELISA)

ELISA examination was performed according to the product instruction. Briefly, a NUNC 96-well plate (Thermo Scientific, MA) was coated with 5 µg/ml of anti-Shh (Sigma Aldrich, MO) or anti-IL-10 (R&D, MN) capture antibody at 4°C overnight, washed with 0.05% PBST and blocked with 5% bovine serum albumin at room temperature for 1 hours, and then incubated with culture supernatants at room temperature for 2 hours. After 5 times rinsing with 0.05% PBST, 2 µg/ml of detecting antibody was added and incubated at room temperature for 2 hours. Following 5 times PBST rinsing, the Biotin-HRP conjugated second antibody was added at a concentration of 1∶5000 at room temperature for 1 hour. Then, o-phenylenediamine dihydrochloride substrate (Sigma Aldrich, MO) was added to develop the signal for detection.

### Histological Examination

For routine histological examination, 4 µm sections of 10% formalin-fixed, paraffin-embedded tissue were prepared and stained with hematoxylin and eosin. All microscopic sections were analyzed by 3 pathologists in a blind fashion. Necrosis, inflammation, and edema were scored from 0 to 3 depending on severity, and a total severity score was calculated (maximum, 9 points).

### Myeloperoxidase Assay and Enzymology Examination

Neutrophil sequestration in pancreas was quantitated by measuring tissue Myeloperoxidase (MPO) activity. MPO was detected according to the protocol of the Chrrmatometric kit (Nanjing Jiancheng Bioengineering Institute, Nanjing, China). Briefly, after weighed, the pancreatic samples were thawed, homogenized in 1 ml of 20 mM phosphate buffer (pH 7.4), centrifuged (10,000×*g*, 10 min, 4°C), and the resulting pellet was resuspended in 50 mM phosphate buffer (pH 6.0) containing 0.5% hexadecyltmethyl- ammonium bromide. The suspension was subjected to four cycles of freezing and thawing and further disrupted by sonication (40 sec). The sample was then centrifuged (10,000×*g*, 5 min, 4°C) and the supernatant was used for MPO assay by utilizing 0.0005% hydrogen peroxide as a substrate. MPO activity was determined by degrading 1 µM of peroxide per minute at 25°C. Results were expressed as units per gram weight (U/g) of wet tissue.

Serum amylase and lipase levels were determined by using an automatic biochemical analyzer (Olympus AU5400, Japan) in the Biochemical Center of Affiliated Hospital of Luzhou Medical College. Serum amylase and lipase levels are expressed in terms of IU/L.

### Statistical Analysis

Experimental values are expressed as mean ± standard error of the mean if not otherwise indicated. Statistical significance was analyzed using SPSS 10.0 software and determined by unpaired Student’s t-tests and one-way analysis of variance. P value <0.05 was considered statistically significant. All results were reproduced in at least three independent experiments.

## Results

### The Expression of the Shh was Increased in the Cerulein-induced Acute Pancreatitis in Mice

**Figure 1:**
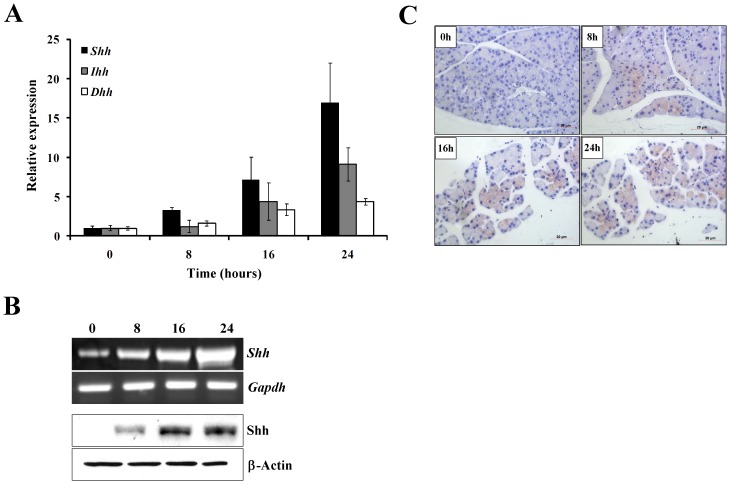
The expression of Shh was upregulated in cerulein-induced mice model of acute pancreatitis in vivo. (**A**). Real time PCR showed the mRNA levels of *Shh, Dhh* and *Ihh* during the development of cerulein-induced mice model of acute pancreatitis. 2 µg of total RNA isolated from fresh tissue was used for reverse transcription, and 1 µl of cDNA was used for qPCR reaction. The *Gapdh* was used as the control. (**B**). RT-PCR (upper two panels) and Western Blot (lower two panels) verified the mRNA expression and protein level of *Shh*. In the DNA electrophoresis 2% of gel was used, in the Western blot 4–10% gradient gel was used, and the *Gapdh* and β-actin were served as control respectively. (**C**). Immunohistochemistry showed the expression of Shh in pancreatic tissue treated with 50 µg of cerulein for time points at 0 hour, 8 hours, 16 hours and 24 hours. Red bar, 20 µm.

There are three hedgehog moleculars known existing in mammalian, the Sonic hedgehog (Shh), the Indian hedgehog (Ihh), and the Desert hedgehog (Dhh), and they all have different expression patterns [7]. To explore whether the expressions of these hedgehog proteins are activated and which one (ones) plays the most important role in acute pancreatitis, we induced the mouse acute pancreatitis model with cerulein and detected the expression level of all these three Hh genes during the acute pancreatitis progression. The mouse model of acute pancreatitis was successfully induced as verified by histological and morphological characteristic (data not shown). In the fresh pancreatic tissue from mice treated with cerulein, we found that the mRNA levels of all three Hh genes were increased actually by real time PCR, but the increasing degrees varied in a large extend. As shown in Figure 1A, the expression of *Shh* increased extremely among these three genes, and the mRNA level increased about 4, 8, and 16 folds at the time points 8, 16, and 24 hours after treatment of cerulein, but the expression of *Ihh* only changed 1, 4, and 7 folds at the same time points. However the expression of *Dhh* had no obvious significance in this pathophysiological process. Using RT-PCR and Western blot, the expression pattern of *Shh* mRNA and Shh protein were confirmed respectively (Figure 1B). At the same time, the Immunohistochemistry was performed to detect the Shh expression in vivo in different time points, and as shown in Figure 1C. The Shh expression kept a low level in the normal pancreatic tissue, but became detectable 8 hours after cerulein treatment, and the expression of Shh protein ascended gradually at 16 hours and 24 hours.

**Figure 2:**
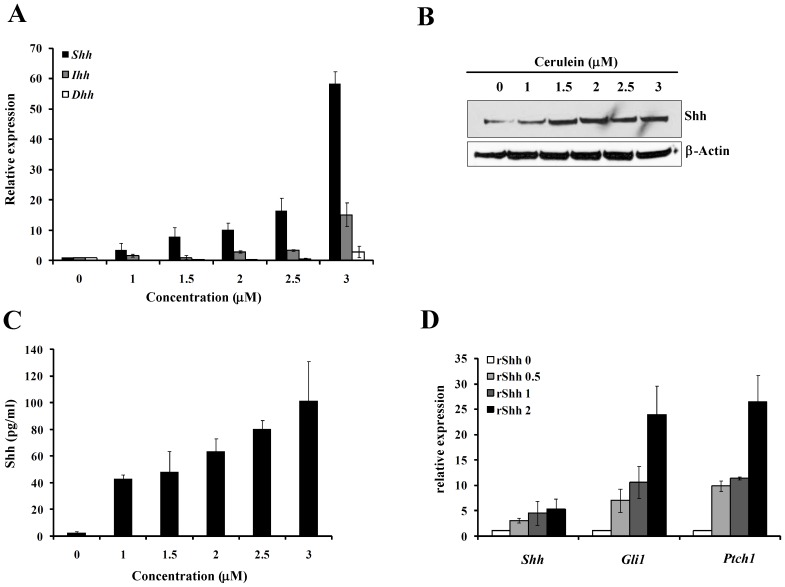
The expression of Shh increased in cerulein-induced pancreatic acinar cell AR42J cells. (**A**)**.** Real time PCR showed the mRNA levels of the *Shh, Dhh* and *Ihh* in rat pancreatic acinar cell AR42J cells treated with different concentration of cerulein for 24 hours, and (**B**) Western blot confirmed the protein expression change, and the *Gapdh* and β-actin ware served as control respectively. (**C**). ELISA detected the secreted Shh in the supernatant of AR42J cell cultures treated with different concentration of cerulein for 24 hours. (**D**)**.** Real time PCR showed the change of hedgehog signaling components mRNA expression, *Shh, Gli1, Ptch1*. Concentration of the recombinant Shh N-terminal was 0, 0.5, 1, and 2 µg/ml. *Gapdh* was used as an endogenous control.

To verify whether the overexpression of *Shh* gene comes from the pancreatic acinar cells, the mRNA and protein expression in a rat acinar pancreatic cell line AR42J treated with cerulein were also examined. The mRNA expression pattern in the pancreatic AR42J acinar cells treated with cerulein for 24 hours behaved almost the same as what we found in cerulein-induced acute pancreatitis tissues in mice, and the predomination of *Shh* expression among the three genes became more obvious, as the mRNA of Shh achieved 60 folds when treated with 3 µM of cerulein (Fig. 2A). The protein expression of Shh was also detected by Western blot, which demonstrated a significant increase of Shh protein (Fig. 2B). Furthermore, both the mRNA and protein expression of Shh were in a dose dependent manner to cerulein concentration.

As the Shh is a secretable factor, we detected the soluble Shh protein in the culture medium of AR42J cells to see whether the upregulated Shh expression is correlative with secretion factor. Using ELISA, we found that AR42J cells without stimulation with cerulein secreted a very low level of Shh, but after cerulein treatment the soluble Shh increased in the supernatant of AR42J cells in a dose dependent manner (Fig. 2C), which illustrated a positive correlation of *Shh* gene expression and autocrine Shh in the cerulein treated pancreatic acinar cells. In the normally cultured pancreatic acinar cells, we added recombinant Shh N-terminal protein to examine whether soluble Shh can activate hedgehog signaling. As shown in Figure 2D, the mRNA expression levels of the hedgehog signaling components, *Shh*, *Gli1*, and *Ptch1* were gradually increased after treated with different dose of rShh. Specially, the mRNA level of *Gli1* and *Ptch1* all augmented as high as 20 folds compared with PBS control, which indicated that the initiation and upregulation of autocrine Shh signaling loop during the progression of acute pancreatitis in mice was established in our study.

### The Shh Signaling Pathway Showed a Protective Effect in the Cerulein-induced Acute Pancreatitis in Mice

**Figure 3:**
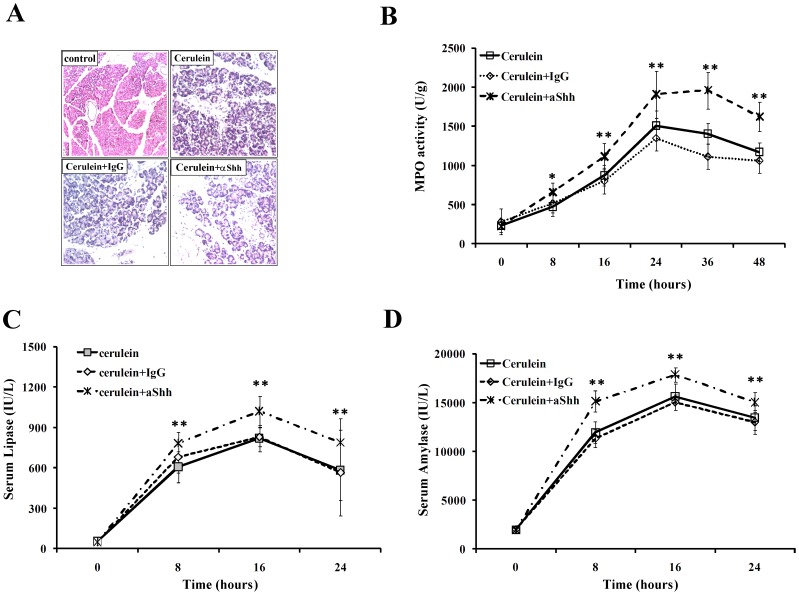
Blockage of Shh aggravates the cerulein-induced acute pancreatitis in mice. (**A**). HE staining of mice pancreatic tissue treated with PBS control, 50 µg of cerulein, combined with 100 µg of IgG control or 100 µg of anti-Shh monoclonal antibody for 24 hours. (**B**). The MPO activities of different groups were measured via neutrophil infiltration. 50 µg of the pancreatic samples were homogenized, and the supernatant was used for MPO activity determined by degrading 1 µM of peroxide per minute at 25°C. Results were expressed as units per gram weight (U/g) of wet tissue. The serum levels of lipase (**C**) and amylase (**D**) were detected by using an automatic biochemical analyzer to indicate the severity of different groups. Serum amylase and lipase levels were expressed in terms of IU/L. There was no significant difference between cerulein group and cerulein with LgG control group in all the MPO activity, serum lipase and amylase level. *, p<0.05; **, p<0.001.

Given the Shh expression was proliferated in acute pancreatitis process, we sought to explore the function of the Shh signaling in acute pancreatitis. Combined with cerulein injection, mice were treated with the anti-Shh neutralizing antibody at the dosage of 100 µg/mouse in order to block Shh signaling and to detect the consequences. Acute pancreatitis development in mice co-treated with Shh neutralizing antibody, but not control IgG was significantly severe than that in cerulein treated only (Fig. 3A). Besides, evaluation of pancreatic myeloperoxidase (MPO) activity and enzymology examination for the inflammation extent were also performed in all the groups. As indicated in our result, the MPO activities in the mice from the Shh neutralization group were all significantly higher than that in mice from cerulein treatment alone group and IgG control group (P<0.05 at time points 8 and 16 hours, and P<0.001 at 24, 36, and 48 hours), and the MPO activity decrudescence in the Shh neutralization group are much more moderate than that in the cerulein or cerulein combined with IgG control group (Fig. 3B). In addition, similar results of the serum lipase and amylase level were observed in these three groups. Increasing of the serum lipase and amylase levels could be detected 8 hours after cerulein treatment, and 16 hours reached their peak value, and then slipped slowly. However, when mice administrated with Shh neutralizing antibody, the serum levels of lipase and amylase were significantly high than IgG control groups, which meant severe pancreatitis induced in mice (Fig. 3C and 3D).

### Primary Mouse Acinar Cells Expression Il-10

**Figure 4:**
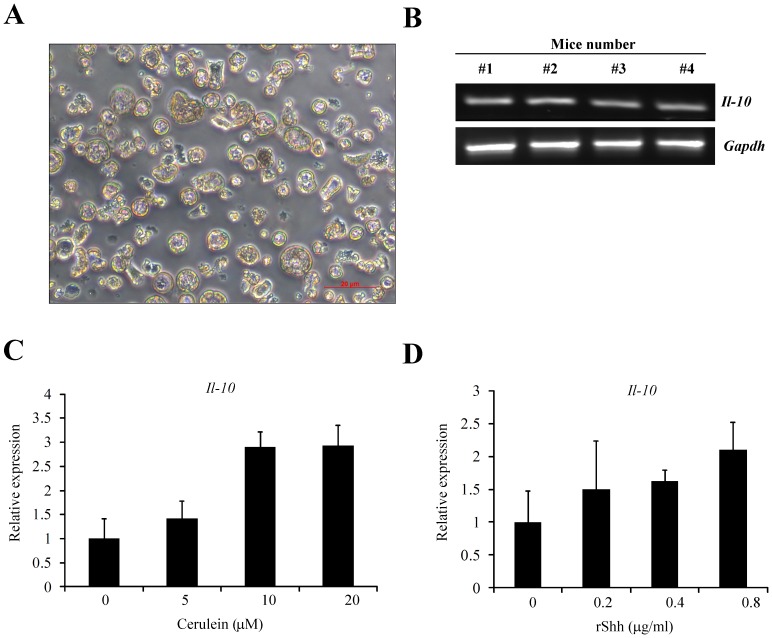
Mouse primary acinar cells express Il-10. (**A**)**.** Morphology of primary acinar cells isolated from mouse pancreas. Magnification, 400×; red scale, 20 µm. (**B**) RT-PCR showed the expression of *Il-10* in mice acinar cells isolated from four different mice. Real time PCR showed the expression of *Il-10* in mice primary acinar cells treated with (**C**) different concentration of cerulein (0∼20 µM) and (**D**) different concentration of recombinant Shh (0∼0.8 µg/ml). *Gapdh* was used as an endogenous control (n = 4).

We isolated the primary acinar cells from mouse pancreas to detect whether the *Il-10* is expressed in the acinar cells in vivo. As shown in Figure 4A, the primary acinar cells were isolated successfully, and the purification and viability was over 96% detected by Countess® Automated Cell Counter (Invitrogen, NY). The expression of *Il-10* was detected by RT-PCR in four mice samples, and after electrophoresized on a 2% agarose gel the 129 bp amplified DNA fragment of *Il-10* was observed, which indicated positive expression of *Il-10* in acinar cells in vivo (Fig. 4B).

To further detected the *Il-10* expression change due to cerulein or recombinant Shh treatment, we added different concentration of cerulein chemical at the concentrations of 0, 5, 10, and 20 µM in the culture medium for 24 hours, and then detected the mRNA expression of *Il-10* by real time PCR. As shown in Figure 4C, the *Il-10* mRNA level increased in a dose dependent manner. Similarly, when the primary acinar cells were treated with the recombinant Shh protein at the concentrations of 0, 0.2, 0.4, 0.8 µg/ml in the culture medium for 24 hours, the *Il-10* mRNA also increased in a dose dependent manner (Fig. 4D).

### Autocrine Shh Alleviates Inflammation in Acute Pancreatitis in Mice by Upregualting IL-10 Expression

**Figure 5:**
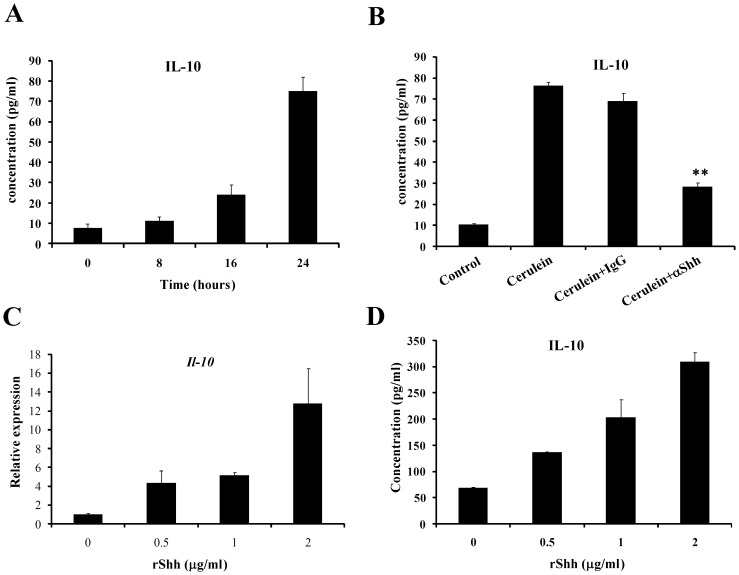
Shh signaling upregualts IL-10 expression in acinar cells. The IL-10 cytokine level in the peripheral blood of mice during the development of cerulein-induced acute pancreatitis was detected by ELISA at (**A**) time points of 0, 8, 16, and 24 hours, and (**B**) in the groups of PBS, cerulein, cerulein combined with IgG control or anti-Shh monoclonal antibody treated for 24 hours. (C) Real time PCR showed that the mRNA level of *Il-10* was upregulated by recombinant Shh N-terminal in a dose dependent manner in AR42J cells. (D) The cytokine level of IL-10 in the culture media of AR42J cells treated with recombinant Shh N-terminal was also detected by ELISA. **, p<0.001.

Sequentially we performed the mechanistic studies to explore the molecular mechanism via which Shh relieves inflammation in acute pancreatitis. It is well known that interleukin-10 (IL-10) limits the severity of acute pancreatitis in mice [21], therefore we detected the IL-10 cytokine level in the peripheral blood of mice during the development of cerulein-induced acute pancreatitis by ELISA. In the normal condition, the basic biological IL-10 cytokine in the peripheral blood of mice is very low, but when mice injected with cerulein, the IL-10 level increased significantly from the 16^th^ hour after treatment (P<0.001), and after 24 hours, the IL-10 level reached 100 pg/ml in the peripheral blood of mice (Fig. 5A), which indicated that IL-10 was excited during the process of acute pancreatitis to confront the inflammation.

To explore whether the increasing IL-10 in the cerulein-induced acute pancreatitis in mice was regulated by autocrine Shh, we detected the IL-10 in the mice from PBS control group, cerulein treated group, cerulein combined with Shh neutralizing antibody, or control IgG groups at the 24 hours after treatment. The IL-10 in the cerulein treated mice was at up to 80 pg/ml, however, once the Shh was neutralized, the IL-10 was significantly downregulated in the peripheral blood (P<0.001), but in the control IgG group there was no significant difference (Fig. 5B).

We performed in vitro experiments to elicit whether the increased IL-10 in mice blood serum was from acinar cells, because during the acute pancreatitis cells from immunity system as well as other tissues are the main source of IL-10 secretion [22], [23]. By addition of recombinant Shh N-terminal protein into the cultured pancreatic acinar cells, the mRNA level of *Il-10* gene was detected by real time PCR. As shown in Figure 5C, the mRNA level of *Il-10* increased in a dose dependant manner according to rShh concentration. Furthermore, the soluble IL-10 in the culture supernatant was also detected by ELISA, and was observed an gradually increasing similar as the mRNA change of *Il-10* gene (Fig. 5D).

### Autocrine Shh Promotes IL-10 Expression in Pancreatic Acinar Cells in a Gli1-dependent Manner

**Figure 6:**
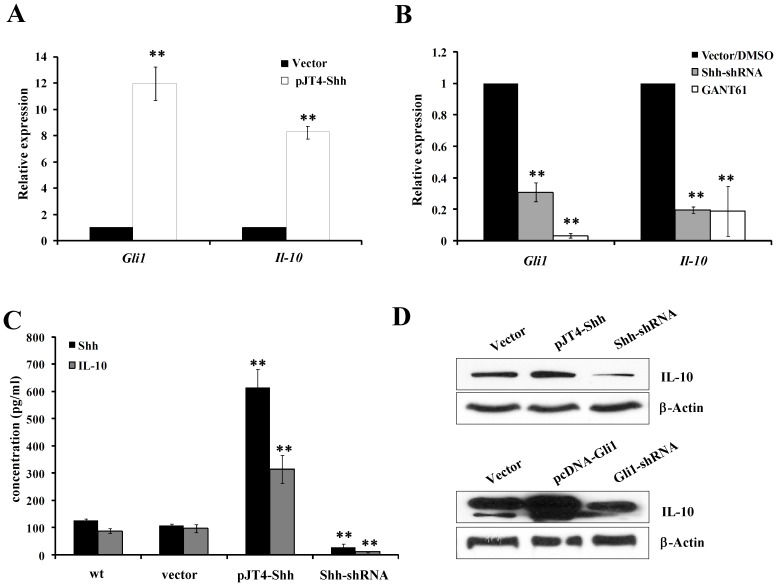
Autocrine Shh promotes IL-10 expression in pancreatic acinar cells in a Gli1*-*dependent manner. (**A**). Real time PCR showed the mRNA levels of *Gli1* and *Il-10* in pancreatic acinar AR42J cells transfected with vector control, Shh expression plasmid pJT4-Shh and Shh specific shRNA. (**B**). Real time PCR indicated the changes of mRNA level of Gli1 and IL-10 in pancreatic acinar cell AR42J when overexpression of vector control or Shh shRNA plasmid, or treated with DMSO mock or 10 µM of the Gli inhibitor, GANT61. 2µg of total RNA isolated from fresh tissue was used for reverse transcription, and 1µl of cDNA was used for qPCR reaction. The *Gapdh* was used as the control. (**C**). ELISA assay showed the Shh and IL-10 cytokine level in the culture supernatant of mice acinar carcinoma AR42J cells transfected with vector control, Shh expression plasmid pJT4-Shh and Shh specific shRNA. (**D**). Western blot confirmed the protein level of IL-10 in AR42J cells transfected with vector control, Shh expression plasmid pJT4-Shh or Shh specific shRNA (upper lane) and Gli1 expression plasmid pcDNA-Gli1 or Gli1 specific shRNA (lower lane).The β-actin ware served as control. **, p<0.001.

We analyzed the mechanism of expression of IL-10 by Shh signaling in the pancreatic acinar AR42J cells. We manipulated the *Shh* gene expression in theAR42J cells with Shh expressing plasmid or Shh specific shRNA to overexpress or knockdown the gene expression. As shown in Figure 6A, the mRNA of *Shh* gene in the pancreatic acinar cells transfected with pJT4-Shh plasmid increased over 1000 folds (data not shown), and accordingly the mRNA expressions of *Gli1*, which is the key transcription factor and also the target gene of the hedgehog signaling augmented over 12 folds (P<0.001). Interestingly, the mRNA expression of *Il-10* was upregulated about 10 folds correspondingly (P<0.01). On the other hand, in the AR42J cells transfected with *Shh* shRNA, or treated with the Gli inhibitor, GANT61, the mRNA of *Gli1* was downregulated significantly, in according with the expression of *Il-10* gene (P<0.01) (Fig. 6B). Moreover, the soluble factors of Shh and IL-10 were also detected in the AR42J pancreatic acinar cells with different expression pattern of Shh. Shh overexpressing AR42J cells secreted significant high level of soluble Shh in the supernatant (P<0.001), as well as the soluble IL-10 level compared with the wild type cells and vector control (Fig. 6C). Using Western blot analysis, the result showed that overexpression of Shh upregulated the protein levels of IL-10, but knockdown of Shh downregulated the protein level of IL-10 compared with the vector control. Similarly, overexpress or knockdown the key transcription factor Gli1 in the AR42J cell also resulted in up or down regulation of IL-10 expression respectively (Fig. 6D).

## Discussion

Hedgehog genes and the hedgehog signaling have been extensively studied in the context of development [7], but there are emerging evidences suggesting that the hedgehog gene expressions are retained or reactivated during adulthood, where they play some roles in oncogenesis, tissue repair, and inflammation in different organs [14], [15], [16]. In the adult pancreas, the expression of the hedgehog signaling molecules, such as Ihh, Ptc, and Smo are localized in the islet cells, ductal cells and tubular complexes, but weakly expressed in normal ductal cells [24], [25], on the contrary the Shh expression is completely absent in normal human pancreatic tissues [26]. Abundant studies have revealed disturbance of expressions of hedgehog components are implicated in some common human pancreatic disorders, including annular pancreas, diabetes mellitus, chronic pancreatitis, and pancreatic cancer. Moreover, hedgehog signaling is also reported to be linked with acute injury and inflammation [27], [28]. However, whether hedgehog signaling plays a role in acute pancreatitis in mice has just come to the fore recently. Volker fendrich *et al* reported that acinar cell regeneration is associated with activation of hedgehog signaling, as assessed by up-regulated expression of Shh and other pathway components using a model of cerulein-mediated injury and repair [17]. In our study, we also confirmed that expressions of all three hedgehogs, Shh, Dhh, and Ihh were increased during the cerulein-induced acute pancreatitis in mice; however, their expression patterns were different. Shh expression was the highest one and kept in a high level within 24 hours in acute pancreatitis, whereas the expressions of Ihh and Dhh were only slightly increased, which revealed in acute pancreatitis the Shh played a predominant role rather than the Ihh and Dhh. Furthermore, we also detected the expression of Shh in the rat pancreatic acinar cell line AR42J, in which the dominant expression pattern of Shh and soluble Shh detected in the culture media of AR42J cells treated with cerulein were the same with that in pancreatic tissue in mice. Thus, we identified the activation and expression of Shh specifically in pancreatic tissue.

Shh ligand is a previously unrecognized anti-inflammatory modulator of the gastroenteritic inflammation, however, it is possible that the early stages of inflammation can activates some protective signaling pathways such as hedgehog to induces cell proliferation or repair the injured pancreas tissue. Nevertheless, Amankulor *et al*, for the first time, established a link between proinflammatory function and the expression of SHH expression during brain injury [29]. But later, Zacharias *et al* reported that acute modulation of Hh signaling resulted in changes in inflammatory pathways, and chronic inhibition of Hh signaling leads to spontaneous intestinal inflammation and death in adult animals, which indicated the Shh hedgehog as an anti-inflammatory factor [16]. In our study, we found that combined with cerulein treatment, mice injected with anti-Shh antibody developed more severe acute pancreatitis than the mice in the IgG and PBS control groups, as the MPO activity, serum lipase and amylase levels in mice blood and tissue evaluation by HE staining from anti-Shh antibody treated group were all significantly severe or higher compared with the control ones. Therefore, we identified the Shh as an anti-inflammatory factor in the cerulein-induced acute pancreatitis in mice.

So far, three principal models have been proposed for the Shh functioning mechanisms in the development and/or oncogenesis: i) ligand-independent signaling, ii) ligand-dependent autocrine/juxtacrine signaling, and iii) ligand dependent paracrine signaling [30]. We found the expression of Shh was mainly localized in the pancreatic acinar cells using Immunohistochemistry staining, which indicated a possible autocrine manner of Shh. To confirm that, we use in vitro experiments by manipulating *Shh* gene expression in the rat acinar cell line AR42J cells with Shh expression plasmid or shRNA plasmid to detect the secreted Shh in the culture supernatant. Interestingly, we found the detectable soluble Shh protein was positively consistency with different *Shh* gene expression manner in AR42J cells. Although, when acute pancreatitis occurs a systematic response will be induced and all Shh-secreting cells may be stimulated to produce Shh to balance the body homeostasis, which means the other sources of Shh, the paracrine Shh, will definitely affect the process of acute pancreatitis, we found autocrine Shh in acinar cell plays an important protective role in the cerulein induced acute pancreatitis, because autocrine Shh activates hedgehog signaling much faster than the long-range paracrine ones so that the sequential protection acts on the acinar cells timely, as the long-range transportation of Shh needs a nanoscale organization [31]. Moreover, the expression of receptors of hedgehog signaling, the patched (Ptch1 and Ptch2) and the Smoothened (Smo) were all expressed (but no Smo expression change) in the acinar cells (data not shown), and addition of recombinant Shh N-terminal protein into the pancreatic acinar cells activated the hedgehog signaling, which illustrated a possible functioning signaling pathway of autocrine Shh in the acinar cells in pancreas in mouse.

Development and progression of inflammation all depends on cytokines [32]. To elicit the detailed mechanism and cytokines involved in Shh mediated anti-inflammatory function, we chose the interleukin-10 (IL-10) as a candidate in our study, as IL-10 is well known for its anti-inflammation effect [33]. Moreover, Mustafa Keceli *et al* studied the effect of IL-10 on acute pancreatitis induced by cerulein in a rat experimental model and found the exogenous IL-10 decreases pancreatic tissue injury induced by cerulein-induced pancreatitis in rats [34]. Warzecha Z *et al* illustrated a new mechanism that protective IGF-1 stimulates production of IL-10 production, the reduction in liberation of IL-1beta and the improvement of pancreatic blood flow so that inhibits development of cerulein-induced pancreatitis [35]. IL-10 was the target gene of Shh signaling in the immune system, as Stewart GA *et al* has reported that the Shh signaling modulates activation of and cytokine production by human peripheral CD4^+^ T cells, and addition of exogenous Shh amplified the production of IL-10 by activated CD4^+^ T cells [36]. But whether IL-10 is a target gene of Shh signaling in cerulein-induced acute pancreatitis has never reported. Based on these findings and theories, we hypothesize that the anti-inflammation effect of Shh in acute pancreatitis in mice is achieved, at least partly, by upregulating IL-10 gene expression. Our data showed that the level of IL-10 in the peripheral blood of mouse with acute pancreatitis induced by cerulein stepped up along with the expression of Shh in the pancreatic tissue, and blockage of Shh signaling attenuated the expression of IL-10 during the development of acute pancreatitis in mice. Moreover, the expression of *Il-10* could be detected in the mouse primary acinar cells, and most importantly, it was regulated by reculein and Shh treatment. In the rat acinar AR42J cells, the expression of IL-10 mRNA and protein as well as the secretion of IL-10 were all in accordance with the overexpression or knockdown of Shh expression. Moreover, overexpression or knockdown the expression of the hedgehog signaling key regulator Gli1 with expression plasmid pcDNA-Gli1 or Gli1 specific shRNA plasmid, otherwise suppression the Gli1 activity by Gli inhibitor GANT61, resulted in upregulation or downregulation of IL-10 expression respectively. In this way, the mechanism of IL-10 as the direct target of Shh signaling pathway to confront acute pancreatitis in mice was elucidated.

In summary, our study on the autocrine Shh is the first demonstration of a new Shh ligand secretion manner and new mechanism of anti-inflammation function via upregulation of IL-10 in the cerulein-induced acute pancreatitis, and would have significant implications for the investigation of Hh pathway signaling in acute pancreatitis as well as lead to the development of more effective therapeutic guidelines for clinical applications.
